# The effect of neonatal vitamin A supplementation on growth in the first year of life among low-birth-weight infants in Guinea-Bissau: two by two factorial randomised controlled trial

**DOI:** 10.1186/1471-2431-13-87

**Published:** 2013-05-23

**Authors:** Sofie Biering-Sørensen, Ane Bærent Fisker, Henrik Ravn, Luis Camala, Ivan Monteiro, Peter Aaby, Christine Stabell Benn

**Affiliations:** 1 Research Center for Vitamins and Vaccines (CVIVA), Bandim Health Project, Statens Serum Institut, Artillerivej 5, Copenhagen S 2300, Denmark; 2 Bandim Health Project, Indepth Network, Apartado 861, Bissau, Guinea-Bissau; 3 Maternidade, Hospital Nacional Simão Mendes, Bissau, Guinea-Bissau

**Keywords:** Neonatal vitamin A supplementation, Low-birth-weight, Growth, Non-specific effects, DTP, BCG

## Abstract

**Background:**

Vitamin A supplementation (VAS) may amplify the effect of vaccines. We therefore investigated if neonatal VAS given with and without Bacille Calmette-Guérin (BCG) vaccine to low-birth-weight (LBW) neonates had an effect on growth in the first year of life. We hypothesised that VAS would be particularly beneficial when provided with BCG.

**Methods:**

We conducted a randomised two-by-two factorial trial in Guinea-Bissau; 1,717 LBW neonates were randomly allocated to VAS or placebo at birth as well as early or the usual postponed BCG vaccination. Anthropometric measurements were obtained at 2, 6, and 12 months after inclusion.

**Results:**

Overall there was no effect of neonatal VAS on growth in the first year of life. By 2 months, VAS tended to have a beneficial effect on weight and head circumference when given with BCG but not when given without BCG (interaction: weight-for-age p = 0.07 and head circumference-for-age: p = 0.06). By 6 months, there was a beneficial effect of VAS on head circumference and weight among children who had not received DTP vaccine 2 months after inclusion (weight: 0.18 (0.00; 0.36) and head circumference 0.27 (0.06; 0.48)), but no beneficial effect among those who had received DTP.

**Conclusion:**

The results support other trials indicating that neonatal VAS does not have consistent effects on childhood growth and if anything the effects seem to be temporary. They also show that the effect may differ by vaccination status, being beneficial when given with BCG at birth and when DTP is delayed.

**Trial registration:**

http://www.ClinicalTrials.gov (NCT00168610) (nct00168610)

## Background

Observational studies have shown that low dietary vitamin A intake and vitamin A deficiency are associated with impaired childhood growth [1-3]. However, a meta-analysis concluded that there was no overall effect of vitamin A supplementation (VAS) to children between 5 and 48 months of age on subsequent growth [4] though it should be noted that one study found that the effect of VAS on growth may differ according to the children’s vitamin A status at the time of supplementation [5]. Only two studies have examined the effect on growth of a single dose of VAS at birth. An Indonesian study including only children with a birth weight ≥ 1500 g found an increase in length at 3 years of age among VAS recipients [6]. Another study conducted in Guinea-Bissau among normal-birth-weight infants (NBW; ≥ 2,500 g) found a positive effect on weight-for-length as long as Bacille Calmette-Guérin (BCG) vaccine was the most recent vaccine. In contrast, there was a tendency for a negative effect among children who had received diphtheria-tetanus-pertussis (DTP) vaccine [7]. None of the trials included children with very low birth weight, who are known to have poorer vitamin A status [8,9].

We have previously hypothesised that VAS amplifies the non-specific effects of routine vaccinations [10,11]. The non-specific effects are the effects of vaccines that cannot be ascribed to the protection against the targeted disease. More specifically, we hypothesised that VAS may amplify the beneficial non-specific effects of the BCG vaccine [10,11]. To test this hypothesis we conducted a trial among low birth weight neonates (LBW; <2,500 g), who normally do not receive BCG vaccine at birth, but only when they have gained weight. In a two-by-two factorial design, LBW neonates were randomly allocated to 25,000 IU vitamin A or placebo and to early BCG or the usual delayed BCG vaccination. When studying the effects on mortality, we found no interactions between BCG and VAS [12,13]. We found no effect of neonatal VAS on infant mortality, but a significant sex-differential effect with a tendency for a beneficial effect in males, but a tendency for a negative effect in females [12]. In the present paper, we have examined the effect on growth of neonatal VAS among LBW children and whether the effect differed by vaccination status (BCG and DTP).

## Methods

### Setting

The Bandim Health Project (BHP) runs a health and demographic surveillance system in the capital of Guinea-Bissau. The present growth study was a part of a two-by-two factorial trial on VAS and BCG at birth which has been described elsewhere [12,13]. In brief, LBW children born at the national hospital from May 2005 to January 2008 were invited to participate. The study was explained to the mothers/guardians in the local language and they received a written explanation in Portuguese. If the mothers/guardians wished to participate, they were asked to sign or put a fingerprint on a consent form. Provided consent, the children were randomly assigned to vitamin A or placebo and to early BCG or the usual postponed BCG. Children assigned to “early BCG” were vaccinated intradermally with 0.05 ml BCG vaccine (Statens Serum Institut, Copenhagen, Denmark). The children who were not assigned to BCG were treated according to local practice and hence not vaccinated at birth. The vitamin A supplement contained 0.5 ml oil with 25,000 IU vitamin A and 10 IU vitamin E; placebo was 0.5 ml oil with 10 IU vitamin E (Skanderborg Apotek, Denmark). The VAS/placebo part of the study was blinded.

### Anthropometrics

Weight, length as well as arm and head circumference were measured by trained field assistants at enrolment and at home visits 2, 6, and 12 months after enrolment. Weight of the undressed child was measured using an electronic scale (SECA Model 835). Length was measured with a measuring mat (SECA Model 210) while the child was lying down. A non-stretching measuring tape (SECA Model 212) was used to measure head circumference. The mid upper arm circumference (MUAC) was measured on the left arm with a non-stretch TALC tape.

If a child was temporarily absent, the assistants would revisit the child one of the following days. Travelling children were revisited at the next round of visits. Children who had moved within Bissau city were located with the help from neighbours. At all follow up visits vaccine information was collected. Most children are breastfed up to 2 years of age [14]; in the present cohort 98% were breastfed by 2 months of age [15].

### Statistical analysis

Measures for weight, length and head circumference were converted to z-scores using the 2006 WHO reference standards [16]. Children were classified as underweight and stunted if they had a weight-for-age z-score < −2 and a length-for-age z-score < −2, respectively. We did not convert MUAC to z-scores since it is not possible to calculate MUAC z-scores for children below 3 months of age and furthermore, we have found that crude MUAC is as good an indicator of mortality risk as MUAC z-score [17]. The effect of VAS on growth was analysed at 2, 6, and, 12 months after enrolment. We used linear regression to compare z-scores at the different time points and calculated relative risks of being stunted or underweight in Poisson regression models with robust standard errors [18]. We adjusted all analyses for the corresponding anthropometric parameter measured at inclusion. As this was a two-by-two factorial trial, all analyses where done for the entire cohort controlled for randomisation to BCG as well as separately in the BCG and No BCG groups. The analyses were also stratified by sex since neonatal VAS might have sex-differential effects [12,19,20].

To study the effect of receiving early or late DTP, we divided the children into two groups; children who had received a DTP vaccine before or on the day of the 2 months visit (Early DTP group) and children who received a DTP vaccine after the day of the 2 months visit (Delayed DTP group) [15] We analysed the effect of VAS on weight-for-age, length-for-age, and head circumference-for-age at 6 months of age adjusted for the corresponding measurement at 2 months of age and for randomisation to BCG in the two DTP groups.

We did not measure vitamin A status at the time of enrolment but as weight is an indicator of vitamin A status [8,9], we examined whether weight group at inclusion modified the effect of VAS on growth.

### Ethical approval

The protocol was approved by the Danish Central Ethics Committee and the Guinean Ministry of Health’s Research Coordination Committee.

## Results

**Figure 1:**
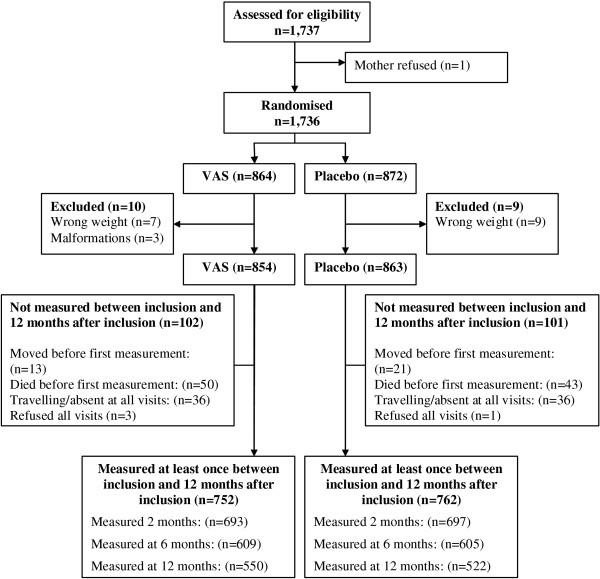
Flow of children through the study.

A total of 1,717 children were randomised to VAS or placebo. At baseline the VAS and placebo groups were comparable [12]. Only 203 children were never measured for growth. The majority had died (n = 93) or were travelling at the time of the visit (n = 72) (Figure 1). These children showed the same distribution of background factors between the VAS and placebo groups (Additional file 1).

Overall there was no difference in weight, length and head circumference between the VAS and placebo groups 2 months after enrolment adjusted for baseline anthropometric measurements and randomisation to BCG (Table 1). The effect of VAS on head circumference-for-age tended to differ with whether given with or without BCG (interaction p = 0.06); the effect of VAS was beneficial when given with BCG (0.16 (0.00; 0.32)), but not when VAS was given without BCG (−0.06 (−0.22; 0.10)). This tendency may have been strongest among boys in whom the beneficial effect of VAS given with BCG was significant (head circumference: 0.37 (0.06; 0.69); head circumference-for-age: 0.26 (0.01; 0.49)). For weight there also was the same tendency towards a beneficial effect of VAS when given with BCG and a negative effect when given without BCG (Interaction: weight p = 0.08; weight-for-age p = 0.07), Neonatal VAS had no effect on MUAC at 2 months after enrolment and no effect on any of the anthropometric parameters at 6 and 12 months after enrolment, neither overall nor separately in the early BCG and No BCG groups (Additional file 2).

**Table 1:** Neonatal vitamin A supplementation (VAS) and anthropometric measurements at 2 months, overall and by BCG-group

		**Overall**				**BCG**				**No BCG**				**Interaction between BCG and VAS**
***All***		**VAS**	**Placebo**	Difference*(CI: 95%)	RR *(CI: 95%)	**VAS**	**Placebo**	Difference** (CI: 95%)	RR ** (CI: 95%)	**VAS**	**Placebo**	Difference** (CI: 95%)	RR ** (CI: 95%)	
	*Number*	693	697			353	350			340	347			
	*Median age in days*	67	66			67	66			66	66			
	Weight, kg	4.25	4.29	−0.01 (−0.07; 0.06)		4.27	4.24	0.05 (−0.04; 0.14)		4.22	4.34	−0.07 (−0.16; 0.03)		**P = 0.08**
	Weight-for-age (WAZ), z-score	−2.13	−2.08	−0.02 (−0.12; 0.09)		−2.09	−2.19	0.08 (−0.07; 0.23)		−2.17	−1.98	−0.11 (−0.26; 0.04)		**P = 0.07**
	Underweight (WAZ < −2)	35%	36%		0.95 (0.83; 1.07)	35%	37%		0.90 (0.76; 1.08)	35%	35%		0.99 (0.83; 1.19)	P = 0.48
	Length, cm	53.0	53.3	−0.11 (−0.37; 0.13)		52.9	53.2	−0.10 (−0.45; 0.25)		53.1	53.4	−0.13 (−0.49; 0.22)		P = 0.88
	Length-for-age (LAZ), z-score	−2.67	−2.53	−0.06 (−0.17; 0.05)		−2.71	−2.58	−0.04 (−0.20; 0.12)		−2.62	−2.48	−0.09 (−0.24; 0.07)		P = 0.66
	Stunting (LAZ < −2)	51%	48%		1.05 (0.93; 1.11)	51%	49%		1.07 (0.94; 1.22)	51%	46%		1.02 (0.90; 1.16)	P = 0.61
	Head circumference, cm	37.4	37.4	0.05 (−0.10; 0.21)		37.5	37.4	0.18 (−0.04; 0.40)		37.3	37.5	−0.08 (−0.30; 0.14)		P = 0.10
	Head circumference-for-age, z-score	−1.32	−1.34	0.05 (−0.06; 0.17)		−1.25	−1.39	**0.16 (0.00; 0.32)**		−1.41	−1.29	−0.06 (−0.22; 0.10)		**P = 0.06**
***Boys***														
	*Number*	311	324			154	167			157	157			
	*Median age in days*	66	66			67	65			66	69			
	Weight, kg	4.38	4.42	0.00 (−0.09; 0.10)		4.39	4.33	0.08 (−0.05; 0.22)		4.37	4.52	−0.08 (−0.22; 0.06)		P = 0.15
	Weight-for-age (WAZ), z-score	−2.29	−2.23	0.01 (−0.15; 0.17)		−2.30	−2.34	0.06 (−0.15; 0.28)		−2.29	−2.11	−0.05 (−0.27; 0.17)		P = 0.54
	Underweight (WAZ < −2)	42%	40%		1.04 (0.88; 1.23)	44%	43%		1.02 (0.81; 1.28)	40%	38%		1.06 (0.83; 1.37)	P = 0.80
	Length, cm	53.4	53.6	−0.04 (−0.41; 0.33)		53.3	53.3	0.10 (−0.42; 0.63)		53.5	53.9	−0.19 (−0.72; 0.34)		P = 0.47
	Length-for-age (LAZ), z-score	−2.91	−2.80	−0.01 (−0.17; 0.15)		−2.98	−2.89	−0.03 (−0.26; 0.19)		−2.85	−2.70	0.01 (−0.21; 0.24)		P = 0.75
	Stunting (LAZ < −2)	61%	55%		1.08 (0.96; 1.21)	60%	60%		1.01 (0.86; 1.18)	62%	51%		1.15 (0.97; 1.37)	P = 0.69
	Head circumference, cm	37.8	37.8	0.10 (−0.13; 0.32)		38.0	37.6	**0.37 (0.06; 0.69)**		37.7	38.0	−0.19 (−0.51; 0.12)		**P = 0.02**
	Head circumference-for-age, z-score	−1.46	−1.45	0.09 (−0.09; 0.26)		−1.37	−1.54	**0.26 (0.01; 0.49)**		−1.55	−1.35	−0.08 (−0.33; 0.16)		**P = 0.06**
***Girls***														
	*Number*	382	373			199	183			183	190			
	*Median age in days*	67	66			68	67			67	66			
	Weight, kg	4.14	4.18	−0.01 (−0.10; 0.08)		4.19	4.17	0.03 (−0.09; 0.16)		4.10	4.19	−0.06 (−0.19; 0.06)		P = 0.24
	Weight-for-age (WAZ), z-score	−2.00	−1.94	−0.04 (−0.18; 0.10)		−1.93	−2.01	0.07 (−0.13; 0.28)		−2.08	−1.87	−0.17 (−0.37; 0.04)		**P = 0.08**
	Underweight (WAZ < −2)	30%	33%		0.87 (0.72; 1.05)	28%	33%		0.82 (0.63; 1.07)	31%	34%		0.93 (0.72; 1.21)	P = 0.49
	Length, cm	52.7	53.1	−0.18 (−0.52; 0.16)		52.7	53.2	−0.32 (−0.80; 0.16)		52.8	53.0	−0.03 (−0.52; 0.45)		P = 0.38
	Length-for-age (LAZ), z-score	−2.47	−2.30	−0.11 (−0.26; 0.03)		−2.51	−2.31	−0.15 (−0.36; 0.06)		−2.44	−2.30	−0.08 (−0.29; 0.13)		P = 0.63
	Stunting (LAZ < −2)	44%	41%		1.03 (0.90; 1.18)	44%	40%		1.06 (0.88; 1.29)	43%	42%		1.00 (0.82; 1.21)	P = 0.80
	Head circumference, cm	37.1	37.1	0.03 (−0.17; 0.24)		37.2	37.1	0.06 (−0.23; 0.34)		37.0	37.0	0.00 (−0.29; 0.29)		P = 0.79
	Head circumference-for-age, z-score	−1.21	−1.24	0.02 (−0.14; 0.17)		−1.15	−1.24	0.07 (−0.15; 0.29)		−1.28	−1.24	−0.03 (−0.26; 0.19)		P = 0.51

*Analyses are adjusted for randomisation to BCG and weight/length/head circumference at inclusion.

**Analyses are adjusted for weight/length/head circumference at inclusion.

Stratified by the children’s DTP status at the 2 months visit, the effect of VAS on weight-for-age and head circumference-for-age was beneficial at 6 months after enrolment if the child had not received a DTP vaccine at 2 months (weight: 0.18 (0.00; 0.36), head circumference: 0.27 (0.06; 0.48)) whereas there was a tendency towards a negative effect if the child had received a DTP vaccine (weight: -0.02 (−0.16; 0.10); head circumference: -0.08 (−0.23; 0.07)) (Table 2). Hence, there was a significant interaction between VAS and DTP for head circumference (interaction: p = 0.01) and a tendency towards an interaction for weight (interaction p = 0.06). When further stratifying on sex, the beneficial effect of VAS on head circumference in the delayed DTP group was most pronounced among girls (difference: 0.40 (0.12; 0.68)) whereas the beneficial effect on weight delayed DTP group was most pronounced among boys (difference: 0.52 (0.24; 0.81)). This resulted in a statistically significant 3-way interaction between sex, VAS, and DTP for weight (interaction: p = 0.01). There was no interaction between VAS and DTP for length at 6 months after enrolment.

**Table 2:** Neonatal vitamin A supplementation (VAS) and anthropometric measurements at 6 months by 2 months DTP-status

	**All**				**Boys**				**Girls**			
	**N**	**VAS**	**Placebo**	Difference* (CI: 95%)	**N**	**VAS**	**Placebo**	Difference* (CI: 95%)	**N**	**VAS**	**Placebo**	Difference* (CI: 95%)
*Weight-for-age at 6 months, z-score*												
DTP at 2 months	715	−1.24	−1.23	−0.02 (−0.16; 0.10)	336	−1.39	−1.31	−0.05 (−0.25; 0.14)	379	−1.11	−1.15	−0.01 (−0.19; 0.18)
No DTP at 2 months	380	−1.67	−1.71	**0.18 (0.00; 0.36)**	154	−1.64	−2.14	**0.52 (0.24; 0.81)**	226	−1.68	−1.39	−0.06 (−0.30; 0.18)
*Length-for-age at 6 months, z-score*												
DTP at 2 months	724	−1.69	−1.71	0.06 (−0.09; 0.22)	343	−1.95	−1.83	−0.08 (−0.32; 0.14)	381	−1.45	−1.59	0.20 (−0.02; 0.41)
No DTP at 2 months	387	−2.13	−2.02	0.03 (−0.19; 0.24)	160	−2.35	−2.30	0.08 (−0.25; 0.41)	227	−1.99	−1.81	−0.02 (−0.30; 0.26)
*Head circumference-for-age at 6 months, z-score*												
DTP at 2 months	723	−0.60	−0.56	−0.08 (−0.24; 0.07)	340	−0.70	−0.59	−0.13 (−0.35: 0.10)	383	−0.52	−0.54	−0.04 (−0.25; 0.17)
No DTP at 2 months	385	−0.61	−0.79	**0.27 (0.06; 0.48)**	160	−0.83	−0.87	0.08 (−0.24; 0.41)	225	−0.47	−0.73	**0.40 (0.12; 0.68)**

*All regression analyses are controlled for randomisation to BCG and weight/length/head circumference at 2 months.

Stratified on weight group at inclusion, there was no beneficial effect of VAS among the different weight groups on weight-for-age, length-for-age and head-circumference-for-age at 2 months (Table 3). Among the smallest girls (weight < 1.5 kg) there was a negative effect of VAS on weight-for-age and head circumference-for-age.

**Table 3:** Neonatal vitamin A supplementation (VAS) and weight, length and head circumference at 2 months by weight at inclusion

	**N**	**VAS**	**Placebo**	Difference* (CI: 95%)	**N**	**VAS**	**Placebo**	Difference* (CI: 95%)	**N**	**VAS**	**Placebo**	Difference* (CI: 95%)
*Weight-for-age, z-score*												
<1.5 kg	86	−4.82	−4.44	−0.31 (−0.73; 0.11)	32	−5.04	−5.07	0.12 (−0.56; 0.81)	54	−4.69	−4.09	**−0.55 (−1.07; -0.02)**
1.5-1.99 kg	360	−2.86	−2.88	−0.10 (−0.27; 0.14)	166	−3.12	−3.16	−0.08 (−0.38; 0.22)	194	−2.64	−2.64	−0.03 (−0.31; 0.24)
2.00-2.49 kg	939	−1.58	−1.58	0.02 (−0-10; 0.16)	434	−1.71	−1.72	0.05 (−0.13; 0.24)	505	−1.47	−1.44	0.00 (−0.17; 0.17)
*Length for age at 2 months, z-score*												
<1.5 kg	86	−5.71	−5.23	−0.31 (−0.83; 0.21)	32	−5.98	−6.26	0.09 (−0.71; 0.90)	54	−5.54	−4.66	−0.51 (−1.18; 0.16)
1.5-1.99 kg	362	−3.34	−3.33	−0.03 (−0.28; 0.21)	168	−3.66	−3.76	0.09 (−0.23; 0.42)	194	−3.06	−2.97	−0.12 (−0.46; 0.23)
2.00-2.49 kg	940	−2.10	−2.01	−0.06 (−0.18; 0.06)	434	−2.34	−2.24	−0.05 (−0.24; 0.14)	506	−1.93	−1.80	−0.09 (−0.24; 0.06)
*Head circumference for age at 2 months, z-score*												
<1.5 kg	86	−3.86	−3.25	**−0.51 (−1.01; -0.01)**	32	−4.29	−3.84	−0.18 (−1.02; 0.67)	54	−3.59	−2.92	**−0.63 (−1.23; -0.02)**
1.5-1.99 kg	362	−1.87	−1.98	0.08 (−0.13; 0.29)	168	−2.02	−2.19	0.13 (−0.19; 0.45)	194	−1.73	−1.82	0.05 (−0.23; 0.33)
2.00-2.49 kg	937	−0.86	−0.94	0.10 (−0.03; 0.22)	432	−0.98	−1.04	0.10 (−0.09; 0.30)	505	−0.76	−0.84	0.08 (−0.08; 0.25)

*All regression analyses are controlled for randomisation to BCG and weight/length/head circumference at inclusion.

## Discussion

There was no effect of neonatal VAS on growth in the first year of life among LBW children with a supposedly low vitamin A status; in particular there was no beneficial effect among the smallest children with a birth weight below 1.5 kg, who if anything had a negative effect of VAS on weight and head circumference. VAS tended to have beneficial effect on weight and head circumference at 2 months of age when given with BCG, but not when given alone. VAS had a beneficial effect on head circumference and weight at 6 months of age in children who had received a late DTP vaccine, but not in children who had received the recommended DTP vaccine before two months of age.

### Consistency with previous studies

The lack of overall effect on infant growth of neonatal VAS found in the present study is in line with the results from previous studies on neonatal VAS [6,7] as well as the results from supplementation later in childhood [4]. A previous trial from Guinea-Bissau that studied the effect on growth of neonatal VAS among NBW infants found a tendency towards a negative effect among girls when DTP was the most recent vaccine [7]. These findings were partly supported by the results from the present study where there was a beneficial effect of VAS on weight and head circumference at 6 months only among the children who had received delayed DTP – though these effects were seen for both sexes.

### Strengths and weaknesses

The present study is the first to assess the effect of neonatal VAS on growth among LBW infants with a presumed poor vitamin A status. We used data from a randomised trial where follow-up was based on home visits. Only 12% were never measured and these children showed the same distribution of background factors between the VAS and placebo groups. It could be speculated that if mortality during follow-up differed between the two groups, then a potential effect of the intervention on growth could be masked, but there was no strong difference in mortality between the VAS and placebo groups. It has previously been shown in the present study population that the delayed DTP group had significantly lower anthropometric measurements indicating that the healthiest children received DTP early [15]. Therefore, we do not know if the tendency for a beneficial effect of VAS in the delayed DTP group is an effect of VAS among the smallest children with the poorest vitamin A status or an effect of VAS and delayed DTP, but it should be noted that there was no general tendency for a better effect in the smallest children. The vitamin A status of the children included in the study was not measured and we used birth weight as an indicator for vitamin A status. Though it would have been optimal to have individual vitamin A status assessed, it has been shown consistently that birth weight is a valid indicator of vitamin A status [8,9].

### Interpretation

We found no indications that neonatal VAS has strong effects on childhood growth. However, we found a tendency towards a positive effect on weight and head circumference when VAS was given with BCG and a negative effect when given without BCG. These results could support our previous hypotheses that VAS amplifies the positive effects of BCG [11]. Furthermore, the timing of the DTP vaccination might influence the effect of VAS on growth at 6 months suggesting that neonatal VAS might amplify a negative effect of DTP or the negative effect of DTP might hinder a beneficial effect of VAS given with BCG. The biological mechanisms behind these effects are unknown.

The beneficial effect of neonatal VAS given with BCG was only seen 2 months after inclusion; 6 and 12 months after inclusion there was no effect of VAS. These results could indicate that the children might have had short term benefits from neonatal VAS correcting a potential vitamin A deficiency at baseline. Later on during the child’s first year of life the vitamin A deficiency might return. However, this would not explain why an initial positive effect of neonatal VAS at 2 months had disappeared at 6 and 12 months. As argued previously it is more likely explained by a negative interaction between VAS and DTP [21]. This is supported by the present study which showed a beneficial effect on weight and head circumference at 6 months after inclusion only among children who had received DTP after 2 months suggesting that the overall no effect at 6 months after inclusion might be caused by the majority of the children receiving DTP before 2 months.

Studies have shown consistently that smaller newborns have poorer vitamin A status [8,9]. However, in our study cohort of LBW infants we found no overall effect of VAS on growth suggesting that even supposedly deficient newborns do not benefit from neonatal VAS.

When we investigated the effect of VAS on mortality, no interaction between VAS and BCG was found, but when looking at the effect on anthropometry we found a tendency towards a different effect of VAS when given with or without BCG. Growth might be a more sensitive outcome than mortality enabling us to discover more subtle differences in effects.

## Conclusions

Overall there was no effect of providing 25,000 IU VAS at birth on growth in the first year of life among LBW children with a presumed low vitamin A status suggesting that neonatal VAS does not impact growth even within this population.

## Competing interests

The authors declare that they have no competing interests.

## Authors’ contributions

CSB and PA designed and initiated the study. ABF, LC, IM, and SBS were responsible for the recruitment and follow-up of participants. SBS was responsible for the statistical analysis with assistance from HR and CSB. SBS wrote the first draft of the paper. All authors contributed to and approved the final version of the paper.

## Pre-publication history

The pre-publication history for this paper can be accessed here:

http://www.biomedcentral.com/1471-2431/13/87/prepub

## Supplementary Material

Additional file 1 **Baseline characteristic by randomisation group among children never measured for growth.** A table showing the difference between the VAS and placebo groups among the children never measured for growth. Click here for file

Additional file 2 **Neonatal Vitamin A supplementation (VAS) and anthropometric measurements at 2, 6 and 12 months, overall and by BCG-group.** A table showing the effect of VAS on MUAC at 2 months and all anthropometric measurements overall and stratified by randomisation to BCG. Click here for file
